# Genetic and phenotypic characterization of a hybrid zone between polyandrous Northern and Wattled Jacanas in Western Panama

**DOI:** 10.1186/s12862-014-0227-7

**Published:** 2014-11-15

**Authors:** Matthew J Miller, Sara E Lipshutz, Neal G Smith, Eldredge Bermingham

**Affiliations:** Smithsonian Tropical Research Institute, Apartado 0843-03092 Panamá, República de Panamá; Department of Ecology and Evolutionary Biology, Tulane University, New Orleans, LA 70118 USA; Patricia and Phillip Frost Museum of Science, 3280 South Miami Avenue, Miami, FL 33133 USA

**Keywords:** Contact zone, Sex-role reversal, Simultaneous polyandry, Introgression, Cline width

## Abstract

**Background:**

Hybridization provides a unique perspective into the ecological, genetic and behavioral context of speciation. Hybridization is common in birds, but has not yet been reported among bird species with a simultaneously polyandrous mating system; a mating system where a single female defends a harem of males who provide nearly all parental care. Unlike simple polyandry, polyandrous mating is extremely rare in birds, with only 1% of bird species employing this mating system. Although it is classically held that females are “choosy” in avian hybrid systems, nearly-exclusive male parental care raises the possibility that female selection against heterospecific matings might be reduced compared to birds with other mating systems.

**Results:**

We describe a narrow hybrid zone in southwestern Panama between two polyandrous freshwater waders: Northern Jacana, *Jacana spinosa* and Wattled Jacana, *J. jacana*. We document coincident cline centers for three phenotypic traits, mtDNA, and one of two autosomal introns. Cline widths for these six markers varied from seven to 142 km, with mtDNA being the narrowest, and five of the six markers having widths less than 100 km. Cline tails were asymmetrical, with greater introgression of *J. jacana* traits extending westward into the range of *J. spinosa*. Likewise, within the hybrid zone, the average hybrid index of phenotypic hybrids was significantly biased towards *J. spinosa*. Species distribution models indicate that the hybrid zone is located at the edge of a roughly 100 km wide overlap where habitat is predicted to be suitable for both species, with more westerly areas suitable only for *spinosa* and eastward habitats suitable only for *J. jacana*.

**Conclusion:**

The two species of New World jacanas maintain a narrow, and persistent hybrid zone in western Panama. The hybrid zone may be maintained by the behavioral dominance of *J. spinosa* counterbalanced by unsuitable habitat for *J. spinosa* east of the contact zone. Although the two parental species are relatively young, mitochondrial cline width was extremely narrow. This result suggests strong selection against maternally-inherited markers, which may indicate either mitonuclear incompatibilities and/or female choice against heterospecific matings typical of avian hybrid systems, despite jacana sex role reversal.

**Electronic supplementary material:**

The online version of this article (doi:10.1186/s12862-014-0227-7) contains supplementary material, which is available to authorized users.

## Background

Hybridization has played a central role in our understanding of speciation (e.g. [1-3]). Studies of hybrid zones can inform our understanding of how environment interacts with genetics and behavior to form species boundaries [4]. The dynamics of hybridization are determined by the degree of genetic exchange among parental species offset by interspecific competitive or antagonistic interactions [5,6]. Among birds, hybridization is quite common, having been documented in nearly 10% of all bird species [1], and over 200 avian hybrid zones have been formally described [3]. Several studies have identified a key role for environmental gradients in maintaining avian hybrid zones [7], with many well-studied hybrid zones centered on ecotones, (e.g.: *Sphyrapicus* sapsuckers [8]), *Callipepla* quails [9], and *Ficedula* flycatchers [10]. Theory establishes environmental differences as a principle mechanism limiting hybridization [11].

Likewise, genetics play an important role in determining the nature of avian hybrid zones. Multi-locus and genome wide studies have shown extensive variation in the degree of introgression across a hybrid zone among different genetic loci (e.g., [12,13]). Price [3] found avian hybrid zones could be clustered into two categories. Those where the parental species were relatively young, as measured by the degree of mitochondrial DNA (mtDNA) divergence, typically had wide hybrid zones, whereas the hybrid zone of relatively old species pairs (again measured by mtDNA divergence) were typically narrow. It remains to be determined whether mtDNA divergence is effectively neutral and simply a proxy for over all genetic incompatibility (e.g. [14]), or may directly cause incompatibilities between mitochondrial and nuclear cellular respiration genes [15,16].

At the same time, recent studies have emphasized that avian behavior should be considered along side environment and genetics as important factors shaping hybrid zone dynamics. This is often observed in cases where the hybrid zone is dynamic, as in western North America where the behavioral dominance of Townsend’s Warblers over Hermit Warblers results in a southward shift of their hybrid zone [17,18], or for *Manacus* manakins in Panama, where female preference for heterospecific golden plumage results in a displacement of sexually selected plumage traits relative to other phenotypic and neutral genetic markers across the hybrid zone [19,20]. The role of behavior in hybrid zone dynamics is obvious, as ultimately, hybrid offspring are produced when an individual mates with a non-conspecific, either intentionally choosing or failing to discriminate against a hybrid or heterospecific mate [21,22].

The relationship between hybridization and polyandry has been well studied, especially in insects [23,24]. These studies have shown that polyandry increases sperm-competition and post-compulatory conflicts among competing genomes. However, this form of polyandry, which is ubiquitous in nature [25], does not consider the special case of social polyandry that includes sex role reversal, i.e. males taking on the majority of parental care in altricious animals. In contrast, most avian hybridization studies focus on either socially monogamous or polygynous/lekking birds, where males compete for a “choosy” female. In these systems, females usually provide greater parental investment in producing eggs and rearing young, and therefore, are more affected by decreased fitness of hybrid offspring [26]. Thus, hybridization occurs when the female lacks access to conspecific males, mistakes species-recognition signals, or in some cases prefers heterospecific traits [22,25-27].

Hybridization has yet to be definitively recorded, let alone studied, among socially polyandrous birds. Classic polyandry, where one female mates simultaneously with multiple males is very rare in birds, occurring in just 1% of all bird species [28]. In most cases, classic polyandry is associated with sex-role reversal, whereby males take on the majority of parental care and females compete for access to males [28,29]. If one extends the argument that the costs of hybridization are greater for females in typical avian mating systems [22], the reversal of sex roles in polyandrous birds should reduce the selective pressures on females against heterospecific mating. Consequentially, this should lead to increased introgression of maternally-inherited genes across the species boundary relative to biparentally or paternally-inherited traits, at least compared to genes underlying similar traits in socially monogamous or polygynous avian groups. This would include mitochondrial DNA, which in most bird systems shows reduced introgression across hybrid zones relative to autosomal markers and most phenotypic traits [30].

**Figure 1:**
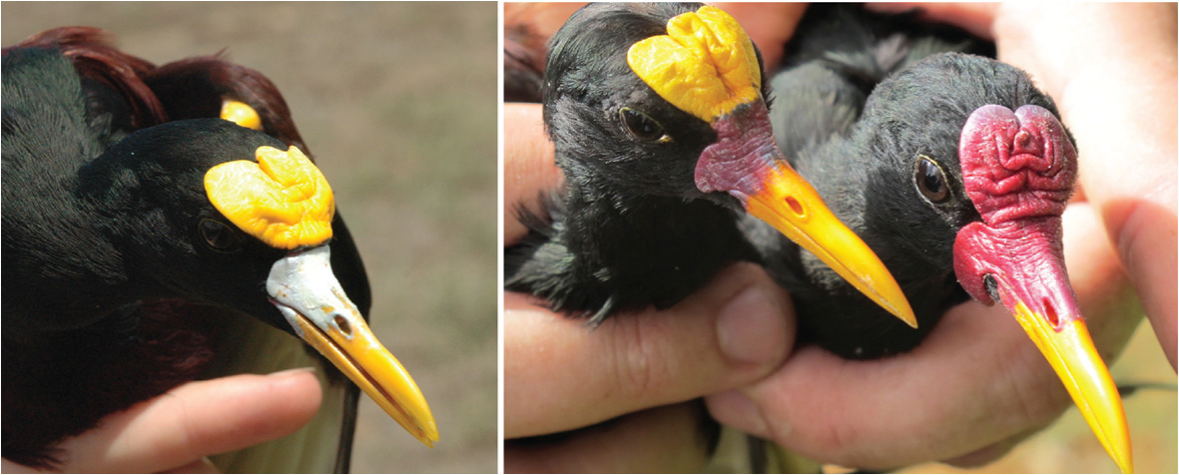
***J. spinosa*** **(Northern Jacana, left), hybrid** ***spinosa*** **X** ***jacana*** **(center), and** ***J. jacana*** **(Wattled Jacana, right).** Photo credits: S. Lipshutz and O. López.

Throughout tropical America, one of the two species of New World jacanas are commonly encountered at most freshwater ponds with extensive floating vegetation. The polyandrous mating systems of both the Northern and Wattled Jacanas (*Jacana spinosa* and *J. jacana*) have been well studied. In both species, females maintain a harem of up to four males by aggressively excluding other females from their territory, and males provide nearly all parental care [31-35]. The ranges of the two species abut in western Panama. A recent comparison of the two species mitochondrial genomes found an average pairwise sequence divergence of only 1.8% in protein-coding regions [36], suggesting that speciation between the two New World jacanas was recent (less than 1 million years before present). *Jacana spinosa* has chestnut-brown dorsal and ventral plumage with a yellow, tri-lobate frontal shield, while *J. jacana* has a red bi-lobate frontal shield and hanging wattles (Figure 1). The subspecies *J. jacana hypomelaena* found in western Panama has all-black plumage, while other subspecies of *J. jacana* found in South America have chestnut-brown plumage.

Here we confirm hybridization between *J. spinosa* and *J. jacana*, describe the hybrid zone based on phenotypic and molecular data, and make a preliminary assessment of the role of genetics, environment, and behavior in mediating hybridization dynamics among polyandrous jacanas. Because this is the first description of a hybrid zone between polyandrous bird species, it provides a useful counterpoint to studies of hybrid zones among avian species characterized by monogamous or polygynous mating systems, and we believe that intensive studies of this system should provide insight into the interaction of genes, environment, and behavior in the avian speciation process.

## Methods

### Sampling

**Figure 2:**
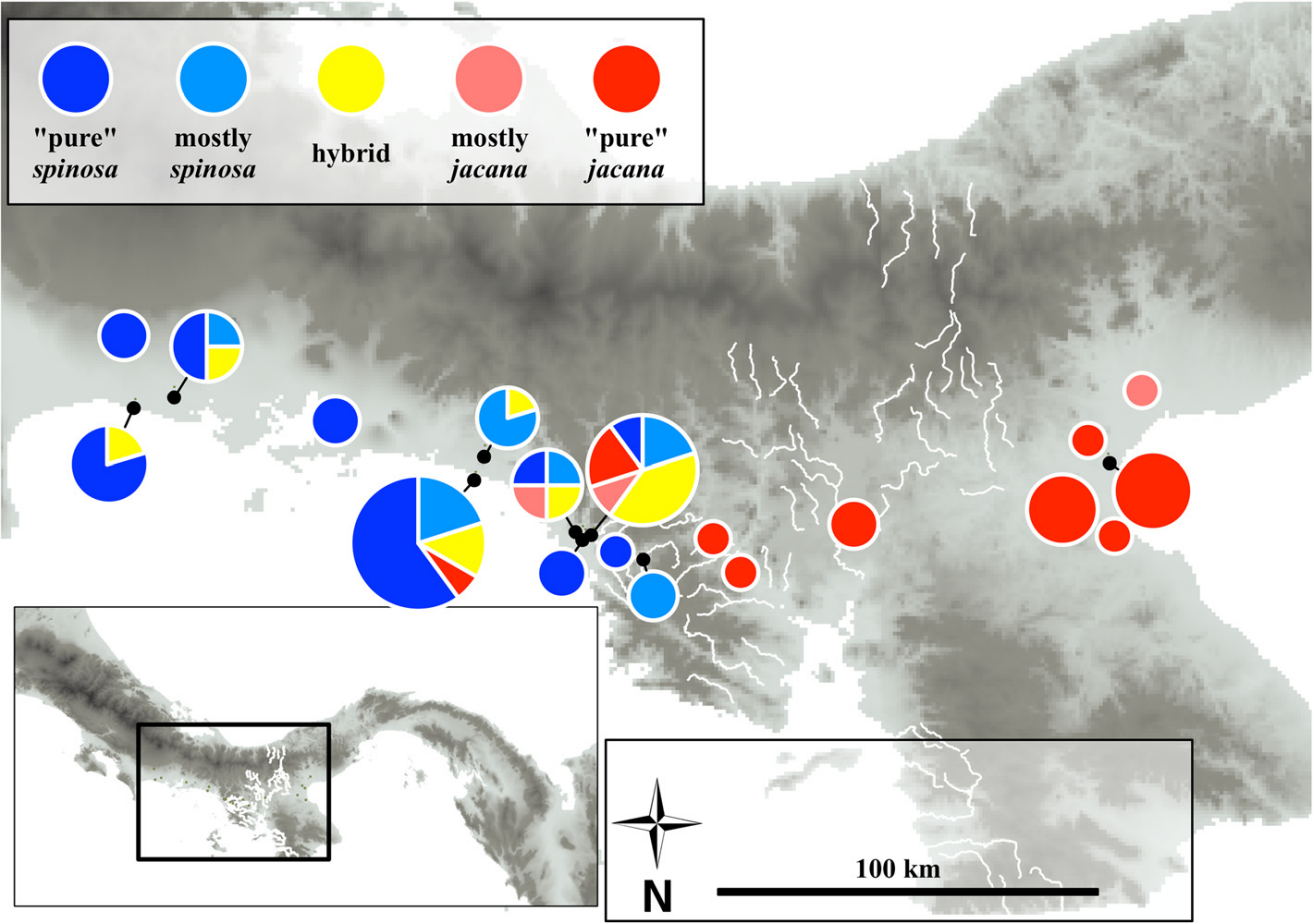
**Distribution of phenotype classes among museum specimens of adult birds across the jacana contact zone in southwestern Panama.** Note the apparent asymmetric introgression of *J. j. hypomelaena* phenotype traits into the range of *J. spinosa*.

An initial series of 13 jacana voucher specimens collected by N.G.S., D. Smith and S. Emlen in June 1994 from the Chiriqui/Veraguas provincial border area in southwestern Panama (see Figure 2) included several birds with obvious intermediate plumage and composite facial ornamentation between *Jacana spinosa*, which ranges from Mexico south to western Panama, and the all-black plumaged *J. jacana hypomelaena*, which is known from central and eastern Panama and adjacent northern Colombia (Figure 1). Other subspecies of *Jacana jacana*, which have chestnut dorsal plumage, are found east of the Andes in tropical South America, and *J. spinosa* is also found in the West Indies. Lacking a large series of jacanas in the research bird collection of the Smithsonian Tropical Research Institute (STRIBC), we focused collecting efforts between December 2010 and February 2012 in this area, sampling opportunistically whenever we encountered jacanas. STRIBC scientific collecting in Panama is done with the prior approval of ANAM, Panama’s environmental authority (permit numbers: SE/A-60- 10, SE/A-137-10, SE/A-96-09, SE/A-44-10, SE/A-66-11, SE/A-2-12), and collecting techniques have been approved by the Smithsonian Tropical Research Institute’s Institutional Animal Care and Use Committee (IACUC permits: 2007-03-03-15-07, 2011-0927-2014-03).

### Morphological comparisons

We scored adult specimen skins maintained in the STRIBC (*N* =67), the American Museum of Natural History (AMNH, *N* =25), and the US National Museum bird collection (USNM, *N* = 10) from Costa Rica, Panama, and Colombia for each of three phenotypic traits: *i*) dorsal plumage, *ii*) ventral plumage, and *iii*) facial shield ornamentation. Following [37], MJM, SEL, and up to three other observers (see Additional file 1: Table S1) scored each bird independently on a scale from 0 – 1, where 0 represented a phenotypically pure *Jacana spinosa*, and 1 represented a phenotypically pure *Jacana jacana* (based on comparisons with skins from outside the contact zone), and we averaged the scores for each trait as the phenotypic hybrid index for each specimen [37]. All five observers scored most specimens, especially those from in and around the hybrid zone. Specimens with phenotypic hybrid indices lower than 0.10 were classified as “pure” *spinosa*, while an index of 0.90 or higher was scored as “pure” *jacana*. A score between 0.10 and 0.25 was classified as “*spinosa*-like”, whereas an index between 0.75 and 0.90 was classified as “*jacana*-like”. Specimens with indices between 0.25 and 0.75 were classified as “hybrids”.

### Genetic analysis

We sequenced a 652 base pair fragment of the mitochondrial *cytochrome oxidase* I gene (COI: the DNA barcode fragment) [38] from 59 Panamanian jacanas, as well as two specimens from eastern Honduras provided for our study by J. Klicka and the Marjorie Barrick Museum. We chose this gene to take advantage of existing sequence data on Genbank for jacanas from diverse geographic regions, which were also added to the dataset (*N* = 10). Laboratory procedures follow standard methods that have been described elsewhere [38]. We attempted but were unable to generate sequence data from toe pad extractions from the Smith and Emlen series of specimens.

The COI data were used to estimate the genetic distinctiveness of the two jacana species and infer the time of speciation between *J. spinosa* and *J. jacana*, and to evaluate the degree to which geography limits gene flow in jacanas (i.e. isolation-by-distance). We generated median-joining network in PopART [39]. We used MEGA [40] to calculate the average distance using the HKY model of molecular evolution in order to compare divergence in jacanas to many other avian species for which genetic distance has been calculated using the HKY model [3].

We tested for the effect of geographic distance on genetic differences (i.e., isolation by distance) via a Mantel test implemented in the program Alleles in Space [41], which compares genetic and geographic distances among individuals rather than populations. For both *J. spinosa* and *J. jacana* mtDNA sequence sets, we correlated uncorrected pairwise sequence divergence with pairwise geographic distance for all possible pairs. Significance of the correlation was determined by comparing the observed correlation to random correlations based on 10,000 permutations.

In order to complement the mtDNA-based analyses of genetic introgression across the jacana hybrid zone, we sequenced two autosomal introns (*gapdh* 11: [42], and *10551*: [43]) for STRIBC specimens collected across Panama. Based on a preliminary assessment of sequence variation from a few samples from each geographic endpoint (outside the hybrid zone) of our sampling design, we identified a single nucleotide polymorphism for each intron: *gapdh* 3- anti-sense position 136 (cytosine-thymine: C-T transition), and *10551* position 153 (adenine-guanine: A-G transition). Positions are relative to aligned sequences of each intron for the *J. spinosa* sample MJM8238 (Genbank accessions: *gapdh*: KM891734, *10551*: KM891735).

### Cline shape analyses

We estimated the shape of the phenotypic and genotypic clines using ClineFit [44], which applies the cline fitting formulas developed by Szymura and Barton [45]. The shape of a cline is determined by three formulas. The first formula (1) defines a symmetric S-shaped curve around the center of the hybrid zone, and relies on two parameters: *c -* for the center of the curve, and *w* - which is 1/maximum slope of the cline, and represents the cline width. Two additional formulae define the cline tails (2,3) and add four additional parameters: *z*_*L*_ and *z*_*R*_, which represent the distance from *c* to a vertical asymptote for the exponential decay of allele frequencies to the left and right sides of the hybrid zone, while *θ*_*L*_ and *θ*_*R*_ define the rates of this exponential decay relative to the shape of the central cline (1).

1 $$ p=\frac{1}{2}\ \left[1+ \tanh \left(\frac{2\left[x-c\right]}{w}\right)\right]; $$

2 $$ p= \exp \left[\frac{4\left(x-\left[c+{z}_L\right]\right)\sqrt{\theta_L}}{w}\right]; $$

3 $$ p=1 - \exp \left[\frac{-4\left(x-\left[c-{z}_R\right]\right)\sqrt{\theta_R}}{w}\right] $$

We compared the fit of a reduced cline model (only formula 1) to that of a model with all six parameters based on the ratio of model likelihoods provided by ClineFit. For two of the three phenotypic traits, the full six-parameter model was a significantly better fit to the data than the reduced two-parameter model, and we subsequently report results of the six-parameter model for all phenotypic and genetic clines. The model also generates a two-unit likelihood (i.e. ln*L*_*max*_ - 2) support region, which is analogous to a 95% confidence limit [46]. Following previous studies [47] we do not report log likelihood support values for our phenotype data as they may violate ClineFit’s likelihood model assuming a genetic model with binomial variance. We established Bebedero, Costa Rica (10.37 N, 85.20 W) as the extreme western-most point (for our cline analyses; the distance from this point to all remaining points was measured as the east–west distance from the 85.20 W meridian as measured in Google Earth. We ran ClineFit with 300 parameter tries per annealing step and 2000 replicates saved, sampling 30 replicates between saves. We tested whether morphological and genetic clines were coincident (i.e. have equivalent centers) and concordant (have equivalent widths) by evaluating whether the parameter estimate of the first cline fell within the estimated support region for the second. We compared the mtDNA cline for jacanas to cline widths from other avian hybrid zones form the literature; methodological details can be found in Additional file 2: Methods S1.

### Ecological niche modeling

To test the possibility that location of the jacana hybrid zone is influenced by environmental variation in western Panama, we generated ecological niche models for both species in Maxent v3.3 [48], using statistical approaches developed in [49], as implemented in the ENMTools v1.3 software package [50]. To do this we generated a set of occurrence points for each species based on our specimen records and supplemented with downloaded data from two public databases (Avian Knowledge Network (http://www.avianknowledge.net) and Ornis (museum specimen records only: http://ornisnet.org). Because occurrence data for both species is extremely biased towards areas of high ecotourism activity, the records were pruned in order to have a uniform distribution of sampled occurrence points across each species range [51]. Ecological niche models were generated in Maxent using the 15 of the 19 standard bioclimatic data layers sampled at 2.5 min resolution from the WorldClim database v.14 [52] trimmed to the current continental distribution of New World jacanas (four layers were found to be highly correlated with other layers [Spearman’s *r* >0.90] s and were excluded from the analysis, *c.f.* [49]. We generated species niche models for three datasets: a) the entire range of *J. spinosa* (239 points), b) the continental range of *J. jacana* (246 points), and c) the range of the all black-plumaged *J.j. hypomelaena* subspecies of *J. jacana* west of the Andes (which are a subset of b), 50 points). For each data set, a species niche model was generated as the average of 100 runs with 25% of the observation points used for model training and the other 75% for model development. The resultant output was a raster file of estimated niche suitability for each species in ASCII format.

We measured the degree to which the estimated niche of each jacana taxon overlapped using three statistics: Schoener’s *D*, a standardized measure of Hellinger distance *I*, [49] and the relative rank *RR* [52], as implemented in ENMTools v1.3 [53]. ENMTools calculated the significance of these statistics using a non-parametric approach by generating null-value distributions of *D*, *I*, and *RR*, which were calculated from 100 pairs of pseudoreplicated datasets that combine occurrence points from both taxa under evaluation. Empirically observed values are then compared to these distributions to determine significance. We used this approach for the comparison of three pairs of modeled niches: *J. spinosa* vs. all *J. jacana*, *J. spinosa* vs. only *J. j. hypomelaena*, and *J. j. hypomelaena* vs. the remaining *J. jacana* (e.g. the chestnut and black plumaged subspecies east and south of the Andes). Finally to attempt to identify which environmental factors were associated with differences in the modeled niches of *spinosa* and *jacana* we compared the mean of all 15 Bioclim parameters for the set of each species occurrence points.

## Results

**Figure 3:**
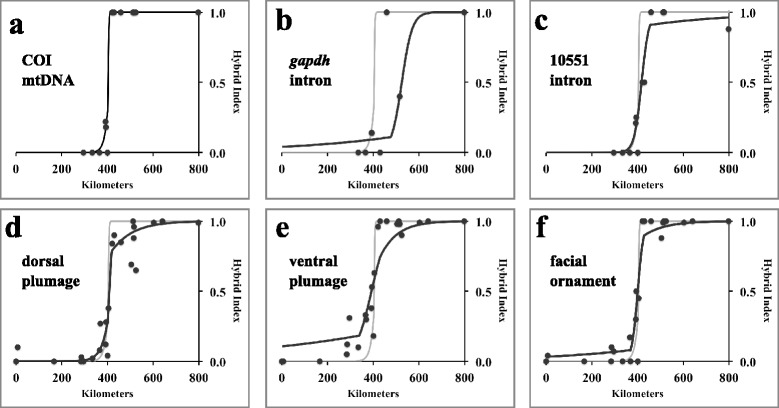
**Fitted geographic clines for genetic markers and phenotypic traits across the jacana hybrid zone.** Points represent frequency of marker and traits at sampled populations. In **b-f)**, grey line shows mtDNA fitted cline for comparison. **a)** COI mtDNA marker; **b)** *gadph* autosomal intron; **c)** 10551 autosomal intron; **d)** dorsal plumage; **e)** ventral plumage; **f)** facial ornament. The cline centers for COI, 10551, and all three phenotypic traits were coincident, whereas the center for *gapdh* was significantly displaced to the east.

In total we scored the phenotypes of 102 adult jacana specimens from Costa Rica, Panama, and Colombia (Additional file 1: Table S1). Phenotypic scores among the observers were highly correlated (Spearman’s *r*: 0.91 – 0.97), and few specimens had scores near the range limit of our classification scheme. Phenotypes scores ranged from 0.00 to 1.00, with the lowest scores in Costa Rica and adjacent western Panama, and highest scores in central Panama east to Colombia (Table 1, Additional file 1: Table S1). Clines fit to all three phenotypic traits had similar centers (average: 402 km east of Bebedero, range: 392 – 408 km). Estimated cline widths for the three phenotypic traits varied from 32 to 142 km (average: 75 km; Table 2). We used the widest value to define the jacana hybrid zone as occurring between 260 and 544 km east of Bebedero. Within this hybrid zone, 16% of specimens were classified as phenotypic “hybrids” and a total of 42% showed some phenotypic signs of introgression relative to parental forms (Table 3). Outside the hybrid zone, all specimens were classified as purely parental. Among those specimens classified as “hybrids”, phenotype scores were significantly biased towards *J. spinosa* (mean score: 0.36 +/− 0.09, one-sample t-test, *t* =4.7, *P* =0.002). East of the hybrid zone center exponential decay tails of our fitted clines quickly reached an asymptote of 1.0. However west of the hybrid center, two of the three fitted clines had shallower exponential decay tails that did not reach 0.0 at Bebedero, suggesting broader, asymmetrical introgression of *jacana* phenotypic traits in the range of *spinosa* (Figure 3).

**Table 1:** **Allele frequency and phenotypic scores by population**

**Population**	**Distance (km)**	***N***_***phenotype***_	***N***_***genotype***_	**mtDNA (%** ***J. jacana*** **haplotypes)**	***gapdh*** **(% C SNPs)**	**10551 (% G SNPs)**	**Dorsal coloration**	**Ventral coloration**	**Facial ornament**
Bebedero	0	2	0	–	–	–	0.00	0.00	0.00
Miravalles	6	3	0	–	–	–	0.10	0.00	0.04
San Isidro	166	2	0	–	–	–	0.00	0.00	0.00
Bugaba	284	2	0	–	–	–	0.00	0.05	0.00
Palo Grande	286	6	0	–	–	–	0.03	0.12	0.10
Orillas del Rio	295	4	5	0.00	–	0.00	0.00	0.31	0.07
Playa Hermosa	334	2	2	0.00	0.00	0.00	0.02	0.10	0.00
Las Lajas	366	15	6	0.00	0.00	0.00	0.08	0.33	0.17
Remedios	369	3	0	–	–	–	0.27	0.30	0.00
Rio Tabasara Delta	392	6	9	0.22	0.14	0.21	0.12	0.38	0.30
La Bromona	394	10	11	0.18	0.14	0.25	0.28	0.53	0.50
Jorones	401	1	1	0.00	–	0.00	0.04	0.18	0.00
El Zapotillo	405	2	0	–	–	–	0.38	0.63	0.45
El Espino	421	1	1	1.00	–	0.50	0.84	0.96	1.00
Tolica	430	1	1	1.00	0.00	0.50	0.90	1.00	1.00
La Corocita	459	2	2	1.00	1.00	1.00	0.85	1.00	1.00
El Rincon	504	4	0	–	–	–	0.69	0.98	0.88
Coclecito	512	2	2	1.00	–	1.00	1.00	1.00	0.99
Cenegon del Mangle	515	1	1	1.00			0.88	0.98	1.00
Aguadulce	516	6	6	1.00	0.40	1.00	0.96	0.98	1.00
Puerto El Gago	524	1	1	1.00	–	–	0.65	0.90	1.00
Gamboa	603	3	0	–	–	–	0.99	0.99	0.99
Tocumen	640	4	0	–	–	–	1.00	1.00	1.00
Aruza Abajo	798	11	6	1.00	1.00	0.88	0.99	1.00	1.00
Barranquilla	1144	4	0	–	–	–	1.00	1.00	1.00
Puerto Berrio	1188	2	0	–	–	–	1.00	1.00	1.00
St. Marta	1209	1	0	–	–	–	1.00	1.00	1.00
Bogota	1226	1	0	–	–	–	1.00	1.00	1.00

Phenotypic and genetic samples and population hybrid index scores across the jacana hybrid zone. Distance is measured as east–west distance from Bebedero, Costa Rica.

**Table 2:** **Geographic cline shape parameters**

**Marker/trait**	**Type**	**Center (c)**	**Width (** ***w*** **)**	***θL***	***θR***	**ZL**	**ZR**
dorsal plumage	phenotypic	408 (n/a)	32 (n/a)	0.08 (n/a)	0.01 (n/a)	28 (n/a)	131 (n/a)
ventral plumage	phenotypic	392 (n/a)	142 (n/a)	0.00 (n/a)	0.23 (n/a)	63 (n/a)	999 (n/a)
facial ornaments	phenotypic	400 (n/a)	52 (n/a)	0.00 (n/a)	0.20 (n/a)	184 (n/a)	999 (n/a)
*gapdh*	autosomal	525 (475–724)	92 (36–637)	0.00 (0.00–0.46)	0.13 (0.00–0.69)	989 (45–999)	450 (38–1000)
10551	autosomal	417 (400–457)	65 (29–181)	0.05 (0.00–0.73)	0.00 (0.000–0.57)	408 (14–1000)	900 (26–997)
COI	mitochondrial	404 (396–425)	7 (4–81)	0.01 (0.00–1.00)	0.55 (0.00–1.00)	15 (6–986)	692 (9–1000)

Geographic cline attributes: centers (*c*) and width (*w*) and two-unit likelihood support limits for three jacana phenotypic traits and two genetic markers estimated in ClineFit [37]; support limits not calculated for phenotypic traits.

**Table 3:** **Jacana phenotype in the hybrid zone**

**Phenotypic classification**	***N***	**Mean hybrid index (95% CI)**	***N***_***males***_	***N***_***females***_	***N***_***unknown***_^**†**^	**Frequency**
“pure” *spinosa*	24	0.03 (0.02 – 0.04)	12	8	4	35%
*spinosa*-like	10	0.13 (0.10 – 0.15)	6	4	--	14%
hybrid	11	0.36* (0.30 – 0.42)	4	6	1	16%
*jacana*-like	8	0.85 (0.82 – 0.88)	4	4	--	12%
“pure” *jacana*	16	0.97 (0.96 – 0.98)	7	9	--	23%
**Total**	**69**		**33**	**33**	**5**	

Distribution of phenotype classes, sexes, and mean hybrid indexes with standard deviation within the phenotypic hybrid zone of jacanas in southwestern Panama. ^†^Specimen labels did not contain information on sex for these specimens. *The mean hybrid index for “hybrid” classified specimens is significantly different from the expected value of 0.50 (see Results).

**Figure 4:**
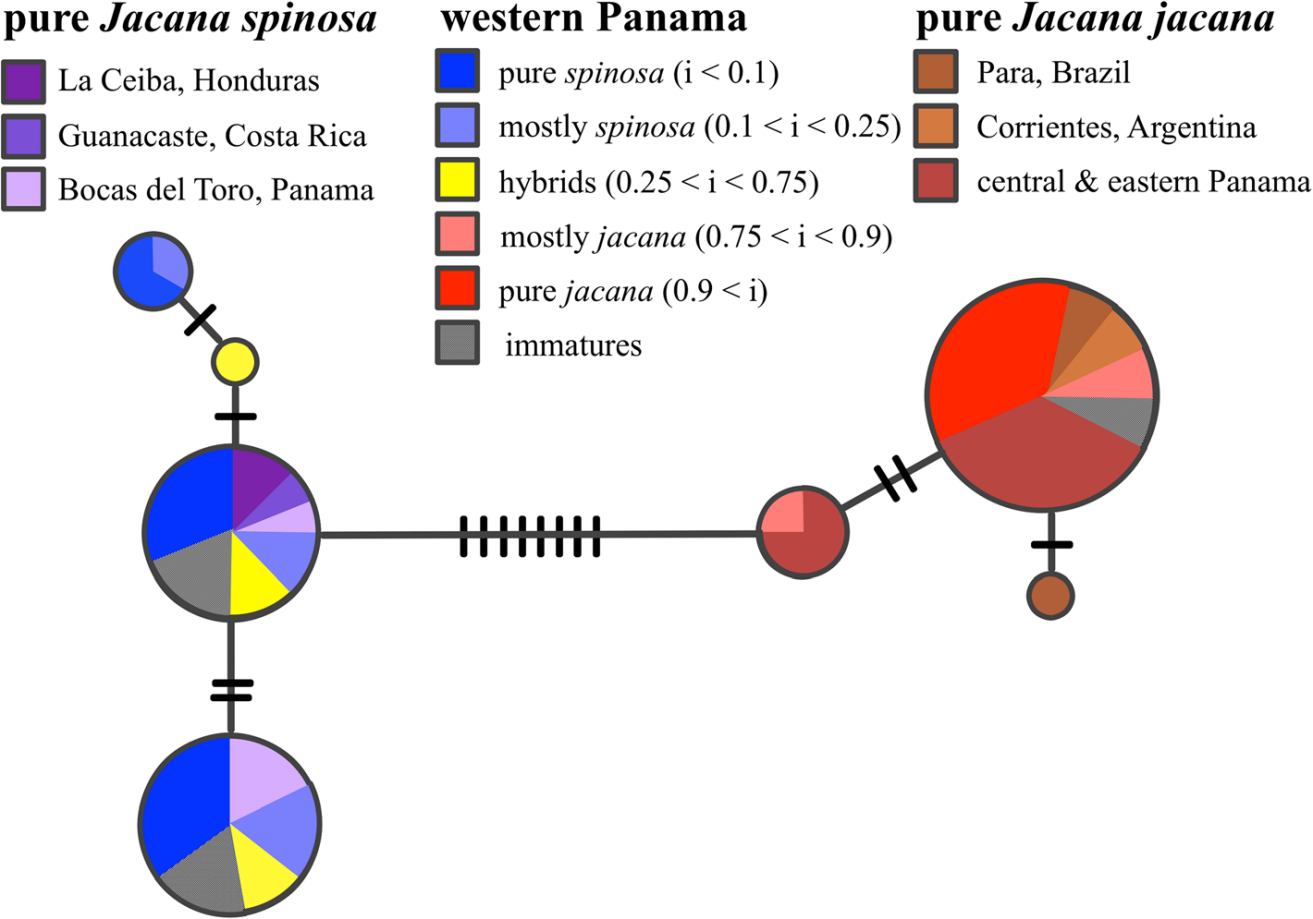
**Unrooted parsimony tree for COI haplotypes of** ***Jacana spinosa*** **,** ***J. jacana*** **, and hybrids throughout their ranges.** Black hatches indicate unobserved haplotypes. Note the lack of geographic structuring within species across distant geographic regions (e.g. Brazil, Argentina and Panama), and that all birds with a hybrid index score greater than 0.3 had *spinosa* haplotypes (see text).

We obtained COI sequences from 59 STRIBC specimens, two from *J. spinosa* specimens from Honduras, and added 11 publicly available COI sequences. Sequence data, trace files and photographs of most STRIBC specimens are available on the BOLD database (dataset: DS-JACANA: dx.doi.org/10.5883/DS-JACANA; and on Genbank: accessions KF919125-KF919186). The COI sequence for MJM 7752 contained one obvious double-peak (A and G) in both the forward and reverse electropherograms at position 511. No other individual in our dataset had an A at this position. As the genetic material was obtained from mitochondrially-rich pectoral muscle, and the sequence shows no other sign of being a nuclear copy, we infer that this is a case of mitochondrial heteroplasmy, and scored that site as A in all further analyses. (Removing this individual entirely from the study had no affect on any result). COI sequences from *J. spinosa* and *J. jacana* sampled outside the hybrid zone formed two monophyletic and species-specific haplotype clusters that showed relatively low pairwise sequence divergence (HKY model corrected distance =1.4%, Figure 4). We found no evidence of isolation by distance in either *J. jacana* or *J. spinosa* COI sequences in our dataset (*J. spinosa*: Pearson’s *r* = −0.14, *P* =0.89; *J. jacana*: Pearson’s *r* = −0.12, *P* =0.94). All phenotypic “hybrids” for which we were able to determine a COI haplotype had a *spinosa* haplotype, which is almost statistically significant, assuming that hybrids should have an equal ratio of *J. jacana* and *J. spinosa* COI haplotypes (n = 5, exact binomial test, *P* =0.063).

Populations of phenotypic *spinosa* as well all individuals classified as “*spinosa*-like” were fixed for cytosine (C) on both chromosomes at position 138 of intron *gapdh*, whereas pure *J. jacana* populations were fixed for thymine (T) at that position; across the hybrid zone the frequency of C vs. T varied clinally (Figure 3, Table 1). Likewise *J. spinosa* were fixed at base 153 of intron 10551 for guanine (G) while in *J. jacana* the frequency of adenine at that position was 88%. Again, across the hybrid zone the frequency of these two bases varied clinally (Figure 3, Table 1).

Cline centers for two of the three genetic markers were coincident with the phenotypic markers, however *gapdh* had a cline center that was significantly east of the other phenotypic and genetic clines (Table 2). Cline widths for the two autosomal markers were less than 100 km and broadly in agreement with the phenotypic cline widths. The estimated cline width for the mitochondrial COI marker was just less than 7 km wide (2 lnL_max_ range: 4 – 81 km). Our finding of such an extremely narrow mtDNA introgression cline was in accord with our observation that populations with mixed *J. jacana* and *J. spinosa* COI haplotypes were found only immediately adjacent to the mouth of the Rio Tabasará (392 km east of cline Bebedero) and La Bramona (394 km east of Bebedero; Figure 2); however, at both these locations *spinosa* COI haplotypes were more frequent than *jacana* COI haplotypes. The easternmost *spinosa* COI haplotype was found 401 km east of the Bebedero. In summary, clines for five of the six markers were coincident, and approximately concordant, although the estimated width for ventral plumage and *gapdh* fell outside the lnL_max_ – 2 range of width values recorded for COI.

**Figure 5:**
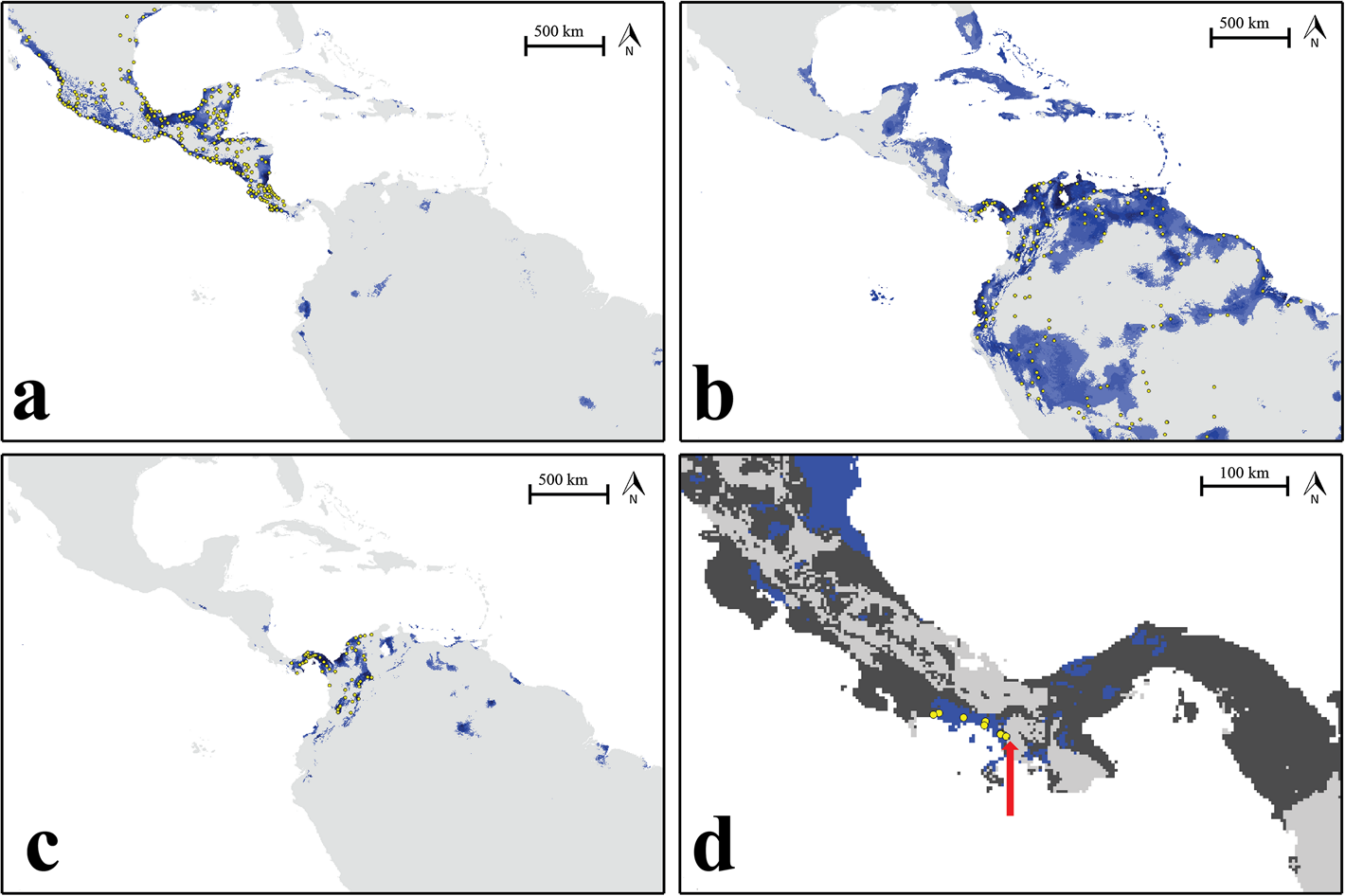
**Maxent-generated species distribution models (SDMs). a)** Northern Jacana (*J. spinosa*); **b)** Wattled Jacana (*J. jacana*); **c)** the *hypomelaena* race of Wattled Jacana; **d)** Close-up of joint SDMs for *spinosa* and *jacana* in their contact zone. In **a-** **c)** yellow circles represent species observations used to generate the models, while blue indicates areas with high habitat suitability in our SDMs (range 0.4 – 1.0). In **d)** dark grey indicates areas where habitats are predicted to be suitable for only one taxon while blue indicates areas where models for both taxa have predicted suitability above 0.4. The red arrow indicates the hybrid zone center, on the eastern edge of a 100 km. wide region of suitable habitat for both species. Yellow circles represent locations where phenotypic hybrid specimens have been collected.

Species distribution models (SDMs) generated in Maxent were better than random predictions and the area under the receiver operating characteristic curve (AUC) approached 1 for all models (*J. spinosa*: 0.958; *J. jacana*: 0.715; *J. j. hypomelaena:* 0.963; *J. jacana – J. j. hypomelaena*: 0.711). The SDMs more or less recovered actual continental distributions; however, our models indicated that the West Indies should be suitable for *J. jacana* (Figure 5b) and not *J. spinosa* when, in fact, it is only the latter taxon that is found on several islands in the Greater Antilles. Tracking actual distributions, the *J. spinosa* SDM indicated high habitat suitability in most of Central America south to western Panama (Figure 5a), while the SDM for the *J. j. hypomelaena* subspecies accurately predicted the current distribution of this subspecies (Figure 5c). However, the SDM for continental *J. jacana* (including *J. j. hypomelaena*) not only included most of its present South American range but also much of the Caribbean lowlands of Central America, which is occupied by *J. spinosa* rather than *J. jacana/J. j. hypomelaena.* It is important to note that all three SDMs show an area of unsuitable habitat in the area of the *J. spinosa* – *J. jacana* contact zone in western Panama (Figure 5a, b, c, d).

Mean Bioclim values between the 239 *J. spinosa* and the 246 *J. jacana* occurrence points were significantly different for 9 of 15 environmental parameters after a Bonferonni control for multiple tests. Six of the nine (BioClim layers 1, 2, 5, 8, 9, 10) measure some aspect of temperature (e.g. annual mean temperature or mean temperature of driest quarter), and on average, *J. spinosa* occurs in warmer (or warmer in a particular season) habitats than *J. jacana* (Additional file 3: Figure S1, Additional file 4: Table S2). The remaining three parameters (BioClim layers 13,15,19) are associated with precipitation; again *J. spinosa* occurs in points with higher rainfall (or higher rainfall in a particular season) relative to *J. jacana* (Additional file 3: Figure S1, Additional file 4: Table S2).

Species distribution models for the various New World jacana taxa all differed significantly in all test metrics (*D*, *I*, *RR*; Additional file 5: Table S3). Most importantly, our results demonstrate that the habitats predicted to be suitable for *J. spinosa* and *J. jacana* are significantly different, regardless of whether we consider all mainland *J. jacana* or just the all-black *J. j. hypomelaena* race. We also found significant differences between *J. j. hypomelaena* and the rest of the continental distribution of *J. jacana*.

## Discussion

Northern (*J. spinosa*) and Wattled Jacanas (*J. jacana*) hybridize where the ranges of the two species meet in western Panama. The narrow hybrid zone appears to be the consequence of the interaction between the behavioral dominance of *J. spinosa* counteracted by unsuitable *J. spinosa* habitat east of the contact zone. Prior to this study, it was unclear whether jacanas even hybridized. Wetmore [54] speculated that some specimens from the region were hybrids, but provided few details. Betts [55] reported a bird with apparently mixed facial ornament and plumage from the Osa Peninsula in southern Costa Rica near the border with Panama, but the hybrid nature of this specimen was rejected by Jenni & Mace [56] who argued that the bird could have been an immature rather than a hybrid, and stated that no conclusive hybrids between the species were known. However, the oldest museum specimen classified as a “hybrid” dates to 1924 and was collected at a location less then 35 km west of the current phenotypic hybrid zone center (and well within the phenotypic introgression zone) suggesting a persistent hybrid zone in southwestern Panama (Additional file 1: Table S1). In general, the nature of hybridization among these species has been obscured by the fact that the *J. j. hypomelaena* subspecies of *J. jacana* occurring in central and eastern Panama has inky-black dorsal plumage whereas the more widespread populations of *J. jacana* in South America have the chestnut-colored dorsal plumage characteristic of all populations of *J. spinosa*. In fact, *J. spinosa* and South American *J. jacana* are best distinguished by facial ornamentation and female size rather than plumage. Our analyses of phenotypic and genetic markers of jacanas across western Panama and beyond firmly establish hybridization between the two species, and provide the first documentation of a persistent hybrid zone in a simultaneously-polyandrous bird species [4]. Using quantitative estimates of phenotypic characters, we found that 16% of adult jacanas in the contact zone could be classified as hybrids, and 46% of the specimens showed some evidence of phenotypic introgression between pure parental forms.

We posit recent speciation between the *Jacana spinosa* and *Jacana jacana*. Assuming a rate of mitochondrial COI evolution of 2% per million years [57], we estimate the two species diverged in the late Pleistocene, around 700,000 years before present, a level of divergence less than most Neotropical bird sister species [58]. In fact, this pairwise divergence is similar to, or shallower than mtDNA divergence between Panamanian populations of several Neotropical lowland birds presumed to represent single species (e.g. *Amazilia* hummingbirds: 1.2 – 2.2% [59]; *Glyphorynchus* woodcreepers: 2.6% [60]; *Mionectes* flycatchers: 3 – 4% [61]; and *Cantorchilus* wrens: 5.6% [62]).

Price [3] demonstrated an inverse correlation between the divergence time of parental species and the width of avian hybrid zones, with young taxa typically having hybrid zones between 100 and 600 km wide. Thus, perhaps the most interesting aspect of jacana hybridization is the relative narrowness of the jacana hybrid zone. Two of the three phenotypic traits were estimated to have clines less than 60 km wide, while the estimated cline for both of the autosomal markers was less than 100 km wide (Table 1, Figure 3a, b). And even allowing the widest cline to define the hybrid zone, jacanas hybridize over a zone that is less than 200 km wide. The mtDNA cline is particularly narrow; our estimate indicates it is only 7 km wide. While studies of avian hybrid zones are common, there have been relatively few estimates of cline geometry using mitochondrial markers. Of the fourteen studies with comparable estimates, the jacana cline is among the narrowest (Table 4).

**Table 4:** **Avian mtDNA cline widths**

**Taxon pair**	**Cline width**	**COI HKY distance**	**Reference**
*Larus glaucescens & L. occidentalis*	1140	0.2%	[63]
*Icterus galbula & I. bullockii*	328	0.1%	[47]
*Dendroica coronata & D. auduboni*	297	0.2%	[64]
*Passerina amoena & P. cyanea*	228	11.0%	[12]
*Sphyrapicus ruber & S. varius*	122	3.3%	[65]
*Strix o. occidentalis & S. o. caurina*	94	0.0%	[66]
*Oporornis tolmiei & O. philadelphia*	88	1.9%	[67]
*Pheucticus melanocephalus & P. ludovicianus*	82	5.1%	[68]
*Amphispiza belli nevadensis & A. b. canescens*	53	n/a	[69]
*Catharus u. ustulatus & C. u. swainsoni*	50	n/a	[70]
*Dendroica townsendi & D. virens*	40	2.0%	[71]
*Pipilo maculatus & P. ocai*	15	3.8%	[72]
*Manacus candei & M. vitellinus*	11	n/a	[73]
*Jacana spinosa & J. jacana*	7	1.4%	*This study*

Estimated mtDNA cline width across avian hybrid zones and average HKY distance between mitochondrial COI sequences. Methodological details can be found in Additional file 2: Methods S1.

Theory establishes that in tension zones cline width is a function of dispersal promoting wider clines countered by selection against hybrids leading to narrower clines [3,11]. Our COI data collected from across both species ranges outside the hybrid zone suggests that dispersal limitation is not a factor in shaping jacana hybrid zones as both parental species maintain gene flow over continental-level geographic scales: *J. spinosa* shared COI haplotypes between western Panama and Honduras, while *J. jacana* had shared haplotypes observed between populations in eastern Panama and southeastern South America (Figure 4), with no evidence of isolation by distance in either species across those sampling regions. This phylogeographic pattern is a consequence of ongoing gene flow and/or recent colonization across considerable geographic distances in both species, and establishes the ability for New World jacanas to maintain gene flow over long distances. The alternative, that both species underwent recent, independent, selective sweeps in their mtDNA is ruled out by low inter-specific divergence and within-species mitochondrial polymorphism. Thus, the narrow zone mtDNA introgression permits strong inference of selection against hybrids in jacanas rather than dispersal limiting the width of the hybrid zone.

The distribution of phenotypic traits and mtDNA haplotypes across the contact zone evidences asymmetry in interspecific interactions and trait introgression. First, birds classified as phenotypic “hybrids” had indices significantly biased towards the *spinosa* phenotype, and all such birds had *J. spinosa* mtDNA, a finding that was almost statistically significant given our modest sample size (*N* =5, *P* =0.06). Likewise all phenotypic “hybrids” were found west of the cline centers for all phenotypic and mtDNA clines (Additional file 1: Table S1), however, because we encountered few birds east of the hybrid zone center, this finding may be due to small sample size. Based on our specimens, *J.* s*pinosa* females have a larger average body mass than *J. jacana* females (154.5 ± 4.6 g versus 134.4 ± 3.9 g, t-test, *t =*3.07, *P =*0.007). Within a species, larger-bodied female jacanas are more likely to hold territories compared to smaller females [31,34,74]. The larger size of *J. spinosa* females may allow them to exert territorial control in mixed-species populations in the hybrid zone. Evidence from a pure population of jacanas suggests that only the largest females are capable of maintaining territories [35]. Our finding that all phenotypic hybrids had *J. spinosa* COI haplotypes is consistent with our hypothesis of largely unidirectional hybridization [27] involving *J. spinosa* females and *J. jacana* males. Differential heterospecific aggression has previously been observed in hybridizing *Anas* ducks [75] and *Dendroica* warblers [17], but in these two cases, differences in aggression have been shown to either increase the number of hybrid matings or expand the range of the more aggressive species.

At the same time, ecological factors appear to be influencing the location of the hybrid zone, and the displacement of *J. spinosa* traits eastward. Previous descriptions of New World jacanas have tended to consider the two species ecological equivalents, and have not identified obvious habitat differences between the species [54]. Nonetheless, we found statistically significant differences between the habitat models for *J. spinosa* and *J. jacana*, and between *J. spinosa* and *J. j. hypomelaena*. On average, suitable habitats for *J. spinosa* are wetter and warmer than suitable habitats for *J. jacana* (Additional file 2: Figure S1)*.* The New World jacana hybrid zone is located at the eastern edge of an ecotone, with habitat suitable for *J. spinosa* north and west of the hybrid zone center (Figure 5a), and habitat suitable for *J. jacana* largely found only to the east of the hybrid zone (Figure 5b, c), although several hundred kilometers north of the hybrid zone, our SDM found suitable *J. jacana* habitat within the range of *J. spinosa*. Most importantly, our SDM indicated that suitable habitat for both species occurs west of the hybrid zone center for 100 km (Figure 5d). Suitable habitat for *J. jacana* is also found several hundred kilometers northward, but it is likely that the competitive advantage of *J. spinosa* makes that habitat inaccessible for *J. jacana.* Thus, we hypothesize that the persistent, narrow jacana hybrid zone may be maintained by the counter-acting forces of a competitive advantage of *J. spinosa* females balanced by the lack of suitable habitat for this species east of the contact zone.

Our finding of a potentially persistent, and stationary hybrid zone is in contrast to other avian hybrid zone where differential introgression results in a moving hybrid zone. In both *Dendroica* warblers [17,18] and *Poecile* chickadees [76,77], asymmetrical introgression and behavioral dominance result in a moving hybrid zone whereby the dominant species shifts the hybrid zone into the range of the subordinate species. In a third case, the moving hybrid zone between *Hippolais* warblers in western Europe appears completely caused by interspecific interactions [78], and is unrelated to the location of preferred habitats based on species distribution models similar to those presented here for jacanas. Similar movement of *spinosa*-like phenotypes and genotypes into the range of *J. jacana* is presumably what would occur in the absence of the ecotone, and given the relatively poor historical (e.g. hybrid specimen) record, it is unclear whether the jacana hybrid zone has moved eastward over recent and/or paleoecological time periods. The one hybrid specimen from 1924 provides some evidence that the hybrid zone has been relatively stationary in its recent history, although the asymmetrical clines for ventral plumage, facial ornaments, and *gapdh* suggest that the hybrid zone center may have moved eastward over recent history. Additional sampling of individuals and more nuclear markers should provide further insights. Likewise, it is possible that climate change will result in shifting the hybrid zone, as has been demonstrated recently for *Poecile* [79] and suggested for *Hippolais* [78].

Finally, the narrowness of the mitochondrial COI cline compared to other jacana markers and traits, and also compared to other bird species (Table 4), was unexpected. At the onset of our study, we hypothesized that the reversal of sex roles in jacanas would reduce the selective pressures on females against heterospecific mating, compared to most birds. This, coupled with the relatively young age of the parental species, should have resulted in increased introgression of maternally-inherited genes such as mtDNA. Instead, we found extremely narrow mtDNA introgression, which suggests strong selection — either directly or indirectly — against the introgression of maternally-inherited traits. Possible hypotheses for extremely narrow mtDNA introgression include mitonuclear discordance (e.g. [16,80]), and/or cryptic female choice (e.g. [81,82]). Emlen et al. [83] demonstrated in central Panama (east of the hybrid zone) that up to 74% of broods cared for by male *J. jacanas* included offspring they did not sire, raising the intriguing possibility that in mixed-species populations in the hybrid zone, *J. spinosa* females maintain mixed-species harems but cuckold *J. jacana* males into raising clutches that are primarily *J. spinosa*.

## Conclusions

In this study, we demonstrated unambiguous evidence for ongoing hybridization between the two species of New World jacana freshwater waders, *J. spinosa* and *J. jacana*, two species that are among the 1% of bird species that are polyandrous and sex-role reversed. We demonstrated that jacanas maintain a narrow, and persistent hybrid zone in southwestern Panama that is coincident for three phenotypic traits, mtDNA, and one of two autosomal markers. We found evidence of asymmetry in the hybridization dynamics: among specimens classified as phenotypically “hybrid”, phenotypic hybrid index scores were significantly biased towards *J. spinosa*, and two of the three hybrid clines had asymmetric shapes. Furthermore, ecological niche models indicate that the two species largely have distinct climatological niches, but that the hybrid zone is centered at the eastern edge of a tract of habitat suitable for both species; eastward, only habitats suitable for *J. jacana* occur. We posit that the stability of the hybrid zone, and its asymmetry, may be maintained by the interactions of the behavioral dominance of larger-bodied *J. spinosa* coupled with the lack of habitats suitable for that species beyond the hybrid zone center. Finally, we provide evidence from mtDNA across the range of both species suggests that dispersal does not limit gene flow within parental species, yet we found that the width of the introgression cline for mtDNA is the lowest quantified for any avian hybrid zone, which we conclude provides strong evidence for selection, which may indicate either mitonuclear incompatibilities and/or cryptic female choice against heterospecific matings.

### Availability of supporting data

Specimen data including age class, sex, collecting locality, museum number and images, as well as COI sequence data are available BOLD database: http://boldsystems.org, under the publically accessible dataset: DS-JACANA [http://dx.doi.org/10.5883/DS-JACANA]. The COI alignment and median-joining network are stored as a single newick file on the Figshare data repository [http://dx.doi.org/10.6084/m9.figshare.1201170].

## Additional files

Additional file 1: Table S1. Specimen data, phenotype classification, genetic data, and hybrid index for Jacana museum specimens. Additional file 2:
**Methods S1.** MtDNA cline width methodology. Additional file 3: Figure S1. Box and whisker plots of mean environmental parameter values for occurrence points of *J. spinosa* and *J. jacana* from 15 BioClim environmental layers. Additional file 4: Table S2. BioClim environmental parameters for *J. spinosa* and *J. jacana* occurrence points. Additional file 5: Table S3. Niche overlap comparisons of New World jacana SDMs.
