# Supplementary material for: Genetic and phenotypic characterization of a hybrid zone between polyandrous Northern and Wattled Jacanas in Western Panama
Source: BMC Evol Biol. 2014 Nov 15;14:227. doi: 10.1186/s12862-014-0227-7 (PMC4237789; doi:10.1186/s12862-014-0227-7)
Supplement: Additional file 2: — Methods S1. MtDNA cline width methodology. [file 12862_2014_227_MOESM2_ESM.docx]

**Additional File 2: Methods S1. MtDNA cline width methodology**

Sequences were downloaded from the public data section of BOLD systems [88] on 26 Aug, 2014. Up to the first 15 sequences were chosen for each taxon. Species level taxonomy followed the BOLD database, for subspecies, range maps were consulted to determine subspecific taxonomy. Sequences were aligned in Geneious 7.06 [89], and between taxa HKY distances were computed in MEGA 6 [41]. We could not assign public sequences unambiguously to *Amphispiza belli ssp*., *Catharus ustulatus ssp*., and *Manacus spp*., so these taxa were excluded from HKY distance analysis.

88. **BOLD systems** [http://boldsystems.org]

89. **Geneious v7.06 created by Biomatters** [http://www.geneious.com]
