# Supplementary material for: Genetic and phenotypic characterization of a hybrid zone between polyandrous Northern and Wattled Jacanas in Western Panama
Source: BMC Evol Biol. 2014 Nov 15;14:227. doi: 10.1186/s12862-014-0227-7 (PMC4237789; doi:10.1186/s12862-014-0227-7)

Additional File 2: Figure S1. Box and whisker plots of mean environmental parameter values for occurrence points of *J. spinosa* and *J. jacana* from 15 BioClim environmental layers. Parameter values with significant differences (after Bonferroni correction) between the two species are bounded by a thick red box (see Additional File 3: Table S2).

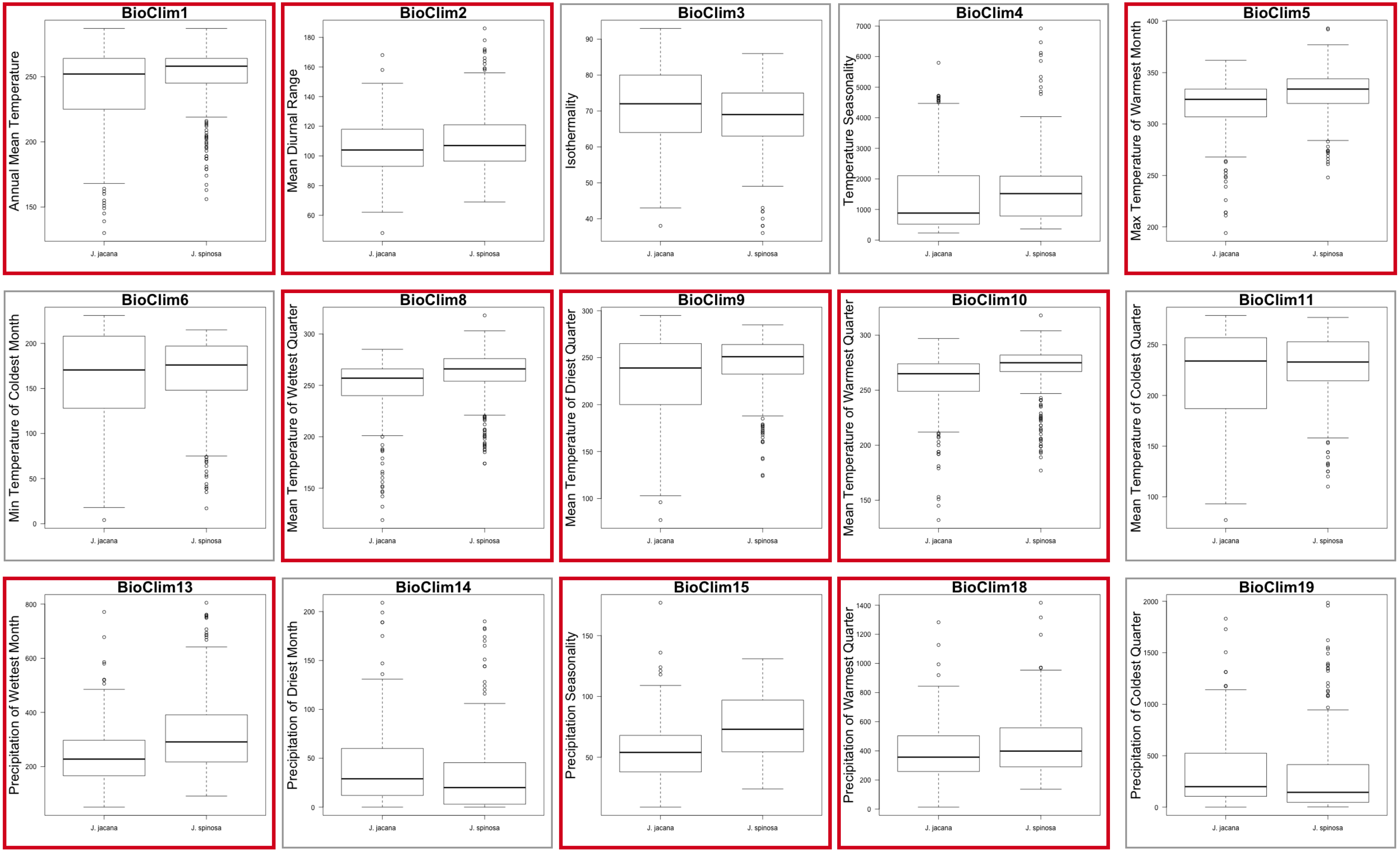

Supplement: Additional file 3: Figure S1. — Box and whisker plots of mean environmental parameter values for occurrence points of J. spinosa and J. jacana from 15 BioClim environmental layers. [file 12862_2014_227_MOESM3_ESM.pdf]
