# Supplementary material for: Genetic and phenotypic characterization of a hybrid zone between polyandrous Northern and Wattled Jacanas in Western Panama
Source: BMC Evol Biol. 2014 Nov 15;14:227. doi: 10.1186/s12862-014-0227-7 (PMC4237789; doi:10.1186/s12862-014-0227-7)
Supplement: Additional file 5: Table S3. — Niche overlap comparisons of New World jacana SDMs. [file 12862_2014_227_MOESM5_ESM.docx]

Additional File 5: Table S3. Results of niche overlap comparisons of New World jacana SDMs. *Obs.*: the observed value of a given test statistic (*D, I,* or *RR,* see Methods); Median, 95% lower, and Min (minimum) refer to the results from 100 pairs of pseudo-replicated datasets generated from the union of both taxa’s sampling points. Significance is determined by comparing *obs*. values to the range of pseudo-replicate values. In this case, for all three taxon comparisons, all *obs.* values were below all 100 psuedo-replicate values, implying a *P*-value ≦ 0.01.

| **Taxa Compared** | ***D*** | | | | ***I*** | | | | ***RR*** | | | |
| --- | --- | --- | --- | --- | --- | --- | --- | --- | --- | --- | --- | --- |
|  | **Obs.** | **Median** | **95% Lower** | **Min** | **Obs.** | **Median** | **95% Lower** | **Min** | **Obs.** | **Median** | **95% Lower** | **Min** |
| *J. spinosa* vs. *J. jacana* | ***0.45*** | 0.84 | 0.82 | 0.80 | ***0.74*** | 0.97 | 0.96 | 0.96 | ***0.60*** | 0.80 | 0.78 | 0.76 |
| *J. spinosa* vs. *J. j. hypomelanea* | ***0.17*** | 0.71 | 0.67 | 0.66 | ***0.36*** | 0.93 | 0.90 | 0.88 | ***0.51*** | 0.79 | 0.76 | 0.75 |
| *J. j. hypomelanea* vs. remaining *J. jacana* | ***0.34*** | 0.82 | 0.78 | 0.76 | ***0.57*** | 0.96 | 0.95 | 0.94 | ***0.57*** | 0.76 | 0.71 | 0.66 |
